# The Allergen-Specific IgE Concentration Is Important for Optimal Histamine Release From Passively Sensitized Basophils

**DOI:** 10.3389/falgy.2022.875119

**Published:** 2022-04-07

**Authors:** Peter Stoffersen, Per S. Skov, Lars K. Poulsen, Bettina M. Jensen

**Affiliations:** Allergy Clinic, Copenhagen University Hospital, Herlev-Gentofte Hospital, Hellerup, Denmark

**Keywords:** IgE, basophil, mast cell, histamine, passive sensitization, allergy

## Abstract

**Background:**

The basophil histamine release (HR) assay can be used for allergy diagnosis in addition to the conventional measurement of allergen-specific IgE (sIgE). Passive sensitization of basophils increases the versatility and allows testing the biological relevance of allergen-induced IgE cross-linking in any serum unbiased by the cellular component. However, not all the patient sera perform equally well and we hypothesized that the absolute level and fraction of sIgE affect the performance. Choosing birch pollen allergy as a model, we investigated the concentration of sIgE needed for successful passive sensitization using soluble- or matrix-fixed Bet v 1.

**Methods:**

Twenty-eight sera with Bet v 1 sIgE [7 sera within each allergy class (1: 0.1–0.70 kUA/L, 2: 0.71–3.50 kUA/L, 3: 3.51–17.50 kUA/L, and 4+: >17.50 kUA/L)] and a negative control serum pool were used to passively sensitize donor basophils, obtained from buffy coat blood (*n* = 3). The cells were incubated (30 min) with a soluble allergen (rBet v 1 from 0.2 to 50 ng/ml), matrix-fixed allergen (ImmunoCAP™ containing recombinant Bet v 1), or phorbol 12-myristate 13-acetate (PMA)/ionomycin mixture (maximal HR) and released histamine was quantified fluorometrically.

**Results:**

The lowest level of Bet v 1 sIgE generating a detectable HR (HR > 10% of maximal release) in all the 3 runs was found to be 1.25 kUA/L (corresponding to allergy class 2, 0.71–3.50 kUA/L). Furthermore, sera from allergy classes 3 and 4+ ascertained a significant reproducible HR: 42/42 vs. 5/21 in allergy class 1 and 15/21 in allergy class 2. Using ImmunoCAP™s containing Bet v 1 as a matrix-fixed allergen system, similar results were obtained where the lowest sIgE concentration mediating an HR was 1.68 kUA/L and 7/7 for both allergy classes 3 and 4+.

**Conclusion:**

The results demonstrate that the IgE titer is strikingly robust in predicting the ability to sensitize basophils and produce a measurable HR.

## Introduction

Basophils are granulocytes found in the blood. They make up <1% of the white blood cells, but are often used in research allergy diagnostic tests due to their resemblance with mast cells, i.e., they release histamine and change surface receptor profile when activated *via* their high-affinity IgE receptors (FcεRI) due to allergen-IgE binding [(1), reviewed in (2)]. Such biological assays often require fresh blood (<24 h old) which can be a challenge in clinical settings. Furthermore, 10–20% of the population have non-releasing basophils (i.e., cells not degranulating), thereby excluding them from these kinds of tests (3).

Passive sensitization (PS) is a technique using basophils from a selected blood donor (confirmed releasing basophils) and serum (IgE) from the patient of interest (4, 5). The basophils have their autologous IgE removed by acidification, making all the FcεRI accessible for the patient serum IgE. These stripped basophils are then incubated with the patient serum allowing binding of IgE to occur. In this way, the IgE fingerprint from the patient will now be found on the donor basophil. PS is, however, not a replacement for serological IgE tests, but it adds a biological factor that can link IgE detection with clinical tests, such as skin prick test (SPT), or even oral food challenge (OFC) and it discriminates if the reactivity is IgE mediated or not (6–9).

Even though PS is a useful technique in advanced allergy diagnosis, it is in the field of allergen characterization where PS has its strength. By keeping the cell source constant, intrinsic mechanisms will not vary and the focus can be kept on the IgE and allergen interaction. Therefore, PS of basophils has been used in evaluating allergen extracts and recombinant allergens according to their biological activity (10–14), risk assessment of biotechnologically-derived products (15), and identification of biologically active food allergens in serum (16, 17).

Different cellular approaches have been used for sensitization besides human basophils (Supplementary Table S1). This includes human mast cells and the rat basophilic leukemia (RBL) cell line expressing the human FcεRI and sometimes also reporter genes (18–25). In addition, the readout can also be cell surface expression of activation receptors (i.e., CD63 and CD203c), as seen in BAT, instead of released histamine (2). However, whether all the sera can be used or if the allergen-specific IgE (sIgE) needs to be in a certain range, as suggested by some publications, is unclear (22, 26).

This study aimed to determine the concentration of sIgE needed to ensure an optimal PS. This was accomplished by the screening of 28 sera from birch pollen-sensitized individuals, employing rBet v 1 as the allergen and basophils from 3 different blood donors and using the basophil histamine release assay (BHRA).

## Materials and Methods

### Selection of Sera

Based on the level of Bet v 1 sIgE, 28 sera [7 sera within each allergy class (1: 0.1–0.70 kUA/L, 2: 0.71–3.50 kUA/L, 3: 3.51–17.50 kUA/L, and 4+: >17.50 kUA/L)] were selected randomly from our serum bank at the Allergy Clinic (see Table 1). The selected sera originated from both mono- and polysensitized individuals. Pooled serum from 120 healthy non-allergic subjects was used as a negative control. This study was approved by the local ethical committee (De Videnskabsetiske Komiteer for Region Hovedstaden), protocol H-3-2010-090.

**Table 1:** Concentrations of Bet v 1 sIgE, total IgE, and % Bet v 1 sIgE in the 28 serum samples and the histamine release (HR) value obtained from the 3 runs of passive HR.

**Sample ID**	**Bet v 1 sIgE (kUA/L)**	**Total IgE (kU/L)**	**% Bet v 1 sIgE**	**Calculated HR-value**		
				**Experiment 1**	**Experiment 2**	**Experiment 3**
A1.1	0.34	4,853	0.01	0	0	43.70
A1.2	0.36	33.7	1.07	0	0	0
A1.3	0.37	46.4	0.80	0	0	1.13
A1.4	0.52	205	0.25	0	0	29.00
A1.5	0.54	37.1	1.46	0	0	0
A1.6	0.64	42	1.52	0	0	1.68
A1.7	0.7	98	0.71	0	0	0.46
A2.1	1.25	241	0.52	0.78	0.83	0.58
A2.2	1.62	29.1	5.57	0	0	0
A2.3	1.68	121	1.39	1.60	0	0.54
A2.4	2.34	339	0.69	3.92	0	0.11
A2.5	2.46	123	2.00	3.54	4.66	3.39
A2.6	2.63	104	2.53	2.13	0	2.47
A2.7	2.64	10.1	26.14	0.81	0.33	0.22
A3.1	6.25	91.6	6.82	15.54	4.74	5.65
A3.2	6.34	32.6	19.45	23.37	32.10	20.03
A3.3	7.25	244	2.97	35.78	16.00	22.73
A3.4	7.46	328	2.27	19.74	5.64	29.59
A3.5	10.3	29.8	34.56	18.72	5.64	12.50
A3.6	11.1	66.8	16.62	25.21	21.85	24.83
A3.7	13.9	70.7	19.66	69.59	28.15	32.99
A4.1	22.2	37.8	58.73	146.63	68.26	90.72
A4.2	26.8	149	17.99	186.52	74.68	164.92
A4.3	28.3	170	16.65	171.98	35.88	151.16
A4.4	33.8	64	52.81	148.60	32.73	129.93
A4.5	37.4	124	30.16	228.15	90.32	237.18
A4.6	45.8	486	9.42	188.39	69.83	82.93
A4.7	56.5	296	19.09	198.28	74.07	154.77

### Measurement of Serum IgE

The level of Bet v 1 sIgE and total IgE was determined by the ImmunoCAP Specific IgE Assay t215 (14-5225-01) and the total IgE assay a-IgE;T (14-4509-01) both from Thermo Fisher Scientific (Uppsala, Sweden). Assays were conducted as described by the manufacturer.

### Selection of Buffy Coat Blood

Fresh anticoagulated buffy coat blood was obtained from anonymous donors at the National University Hospital Blood Bank (Copenhagen, Denmark). To obtain a blood donor with highly responding basophils but without an allergic profile (no reaction to common allergens), the blood was screened as outlined: in a polypropylene tube, 1 ml of buffy coat blood was washed by adding 9 ml of pipes buffer (Hospital Pharmacy, 9.3 mM pipes, 0.14 M sodium acetate, 5.0 mM potassium acetate, 0.60 mM calcium chloride, 1.1 mM magnesium chloride, and adjusted to pH 7.4 with 1 M Tris) and centrifuged at 600 × g for 10 min at room temperature (RT). The supernatant was removed and the buffy coat was resuspended in pipes buffer to a total volume of 3 ml. The cell suspension was then added in duplicate to dilutions of stimulant and basophil histamine release (HR) was determined by the glass fiber method (RefLab, Copenhagen, Denmark): 25 μl of cell suspension was added to a 96 well glass fiber-coated plate containing 25 μl of dilutions of anti-IgE (KPL Inc., Gaithersburg, Maryland, USA, final concentrations: 1,000, 330, 110, 37, 12, and 4 ng/ml), recombinant Bet v 1 (Biomay AG, Vienna, Austria, final concentrations: 50, 17, 6, 2, 0.6, and 0.2 ng/ml), and phorbol 12-myristate 13-acetate (PMA)/calcium ionomycin mixture (Sigma-Aldrich, final concentrations: 1.6/6.7 μM, 0.53/2.2 μM, and 0.18/0.73 μM) or a standard panel of 10 inhalant allergens (grass, birch, mugwort, cat, dog, horse, *Dermatophagoides pteronyssinus, Dermatophagoides farinae, Cladosporium*, and *Alternaria*) and 10 food allergens (milk, egg, wheat, peanut, hazelnut, kiwi, cod fish, shrimp, celeriac, and soy) [Screening plate (RLA217), RefLab]. Cells were incubated at 37°C for 30 min. Subsequently, cells were removed from the plate by washing in deionized water followed by incubation with 0.4% sodium dodecyl sulfate solution (RefLab) at 37°C for 10 min. Plates were washed again and 75 μl 3.7 mM o-phthalaldehyde (RefLab) in 50 mM sodium hydroxide (Hospital Pharmacy) was added. After 10 min, 75 μl 0.59% perchloric acid (Hospital Pharmacy) was added and the released histamine was quantified fluorometrically using the HistaReader 501 (RefLab). Buffy coat donors were selected when basophils elicited an anti-IgE-induced HR of more than 50 ng/ml histamine released (>30% release), but with no HR response to the common allergens or rBet v 1. Buffy coat blood was added recombinant human interleukin 3 (R&D Systems Inc., Minneapolis, Minnesota, USA) to a final concentration of 10 pg/ml and kept at 8–10°C overnight.

### Passive Sensitization

The procedure of PS was carried out without isolation of basophils. In a polypropylene tube, 5 ml of buffy coat blood was mixed with 45 ml of physiologic saline and centrifuged at 1,000 × g for 10 min. at 11°C. The blood cells were resuspended in a 45 ml cold stripping buffer (0.14 M sodium dihydrogenphosphate and 5.0 mM potassium chloride, 4°C, pH 3.55) to remove autologous IgE and immediately centrifuged at 1,000 × g for 10 min at 11°C. Subsequently, the blood cells were resuspended in 45 ml pipes buffer, centrifuged at 1,000 × g for 10 min at 11°C, and cells were resuspended in pipes buffer to a total volume of 5 ml. In total, 1 ml of this cell suspension was added to 125 μl serum and allowed to incubate at 37°C for 1 h in a sealed polypropylene tube. After incubation, the cell suspension was diluted with pipes buffer to a total volume of 3 ml.

### Passive Sensitization Basophil Histamine Release Assay

Basophil histamine release assay (BHRA) was performed using the glass fiber method and 25 μl of the cell suspension of the PS basophils which were stimulated with anti-IgE, recombinant Bet v 1 (rBet v 1) or PMA/ionomycin as described in “Selection of buffy coat blood.” Basophil HR was expressed in percentage of the PMA/calcium ionomycin-induced maximal HR or as HR-value as indicated in the text.

Background values were calculated from the negative control serum pool as “mean HR + 3 × SD,” where mean HR denotes the mean of all dilution points. Only HR values higher than background were used.

CDsens was calculated as the inverted rBet v 1 concentration eliciting 50% of maximum response (1/2max) times 100. If 1/2max could not be determined, CDsens was set to 0. Onset refers to the concentration corresponding to the intercept between the curve and the background. If the curve was above background for all the rBet v 1 concentrations, no value was given. Ymax was defined as the maximum HR. If Ymax was below the background, the value was set to 0. The area under the curve (AUC) was calculated using GraphPad Prism version 9.3.1 for windows. If no curve exists (HR below background), the AUC was set to 0.

### Histamine Release Using Matrix-Fixed Allergen

ImmunoCAP™s containing recombinant Bet v 1 (t215), anti-IgE (assay control) (14-4417-01 (a_IgE;S), or streptavidin (background) (14-5320-01 (o212) (Thermo Fisher Scientific) was placed in a 96-well filter plate where the filter was removed (“ImmunoCAP plate”). ImmunoCAP™s were washed using 10 × 200 μl pipes buffer. Buffer was drained by suction, using an Aurum™ Vacuum Manifold (Bio Rad, Copenhagen, Denmark) and residual buffer was removed by centrifugation (600 × g for 10 s at RT). PS basophils were added to each immunoCAP™. For maximal HR, the cell suspension was first mixed with PMA/calcium ionomycin (1.6 /6.7 μM) and then added to a streptavidin immunoCAP™. The ImmunoCAP plate was then placed on top of a 96-well V-shaped microplate (“Collection plate”) and this “sandwich” was incubated for 30 min at 37°C in a preheated moisture chamber. After incubation, 100 μl of pipes buffer was added to each immunoCAP™ and incubated for 5 min at RT. The ImmunoCAP plate + collection plate sandwich was centrifuged at 300 × g for 5 min at 20°C to recover all the cell suspension into the collecting plate. Released histamine was measured in 50 μl of the supernatant using the glass fiber method, as described in “Selection of buffy coat blood.” Results were corrected for background and considered positive if HR > 5% (negative control serum pool).

### Statistics

Pearson correlation coefficients were calculated using GraphPad Prism 6.00 and 9.3.1 for Windows, GraphPad Software, La Jolla, California, USA, www.graphpad.com.

## Results

### Impact of Specific IgE Concentration for Optimal Passive Sensitization

**Figure 1:**
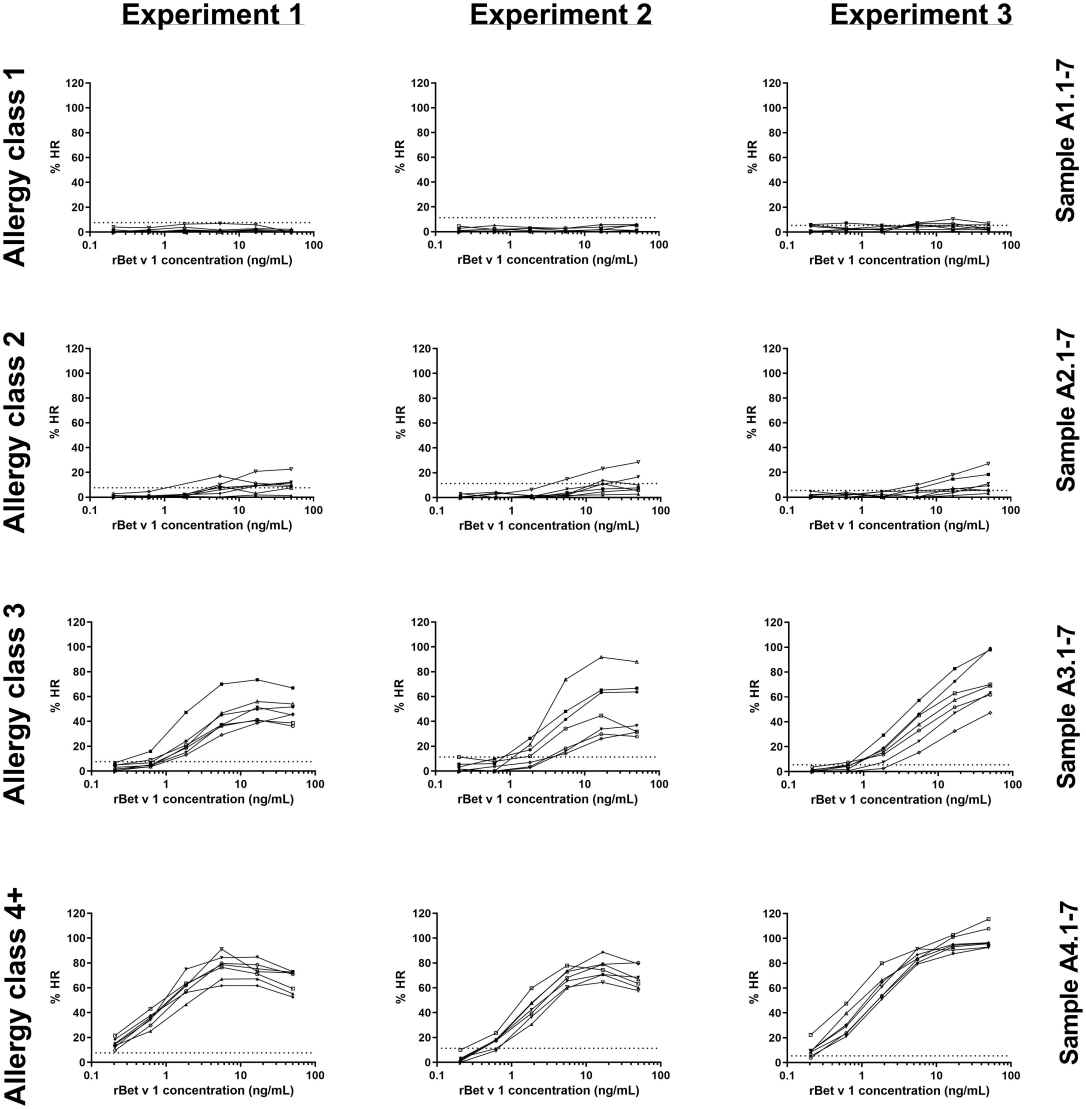
rBet v 1-induced histamine release (HR) curves for 28 sera in experiments 1, 2, and 3. Histamine release is expressed as %HR of the PMA/ionomycin response. Sera are shown in their respective allergy class and each experiment represents a cell donor. Numbers refer to sample ID (Table 1). Dotted horizontal lines indicate background values obtained from the negative control serum pool (8, 11, and 6% for experiments 1, 2, and 3, respectively).

The 28 sera were used for PS of blood donor basophils and the cells were subsequently stimulated with rBet v 1 or PMA/ionomycin. In total, three identical experiments were conducted using a new blood donor for each experiment. As seen in Figure 1, allergy class 1 sera were very poor at mediating an HR and we only saw a positive response from five sera in the third experiment (5/21 sensitizations with allergy class 1 sera). Using allergy class 2 sera only a weak response to the highest concentrations of rBet v 1 was found with 15/21. Allergy class 3 sera constitute a transition from the weak responses elicited by allergy class 2 sera to the strong responses seen with allergy class 4+ sera. Both with allergy classes 3 and 4+ sera 21/21 sensitizations mediated an HR response. Differences in curve patterns between experiments are associated with the different cell donors. Overall, the lowest concentration of sIgE to mediate HR was found to be 1.25 kUA/L, however, to obtain 100% success, sera from allergy classes 3 and 4+ should be used.

### Correlation Between Histamine Release and Specific IgE Using the HR-value

To circumvent the need for subjective interpretation of the HR dose-response curves, we established the HR-value. This is the accumulated weighted area under the curve, as it takes into account both the curve height (reactivity) and the location on the x-axis (sensitivity). In brief, y-values (% HR) above background level (mean HR + 3 × SD of the negative sample) were multiplied by their corresponding inverted concentration and then added up. The HR value was calculated according to the formula:


$$\begin{array}{l} HR-value=C_{6}^{-1}\times Y_{6}+C_{5}^{-1}\times Y_{5}+C_{4}^{-1}\times Y_{4}+C_{3}^{-1}\times Y_{3}\\ \;+C_{2}^{-1}\times Y_{2}+C_{1}^{-1}\times Y_{1} \end{array}$$

Where C_6_-C_1_ denotes the rBet v 1 concentration in ng/ml (0.2–0.6–2–6–17–50) and Y_6_-Y_1_ is % HR. If the HR ≤ background, the HR value was set to 0. Calculated HR values for each experiment are given in Table 1.

**Figure 2:**
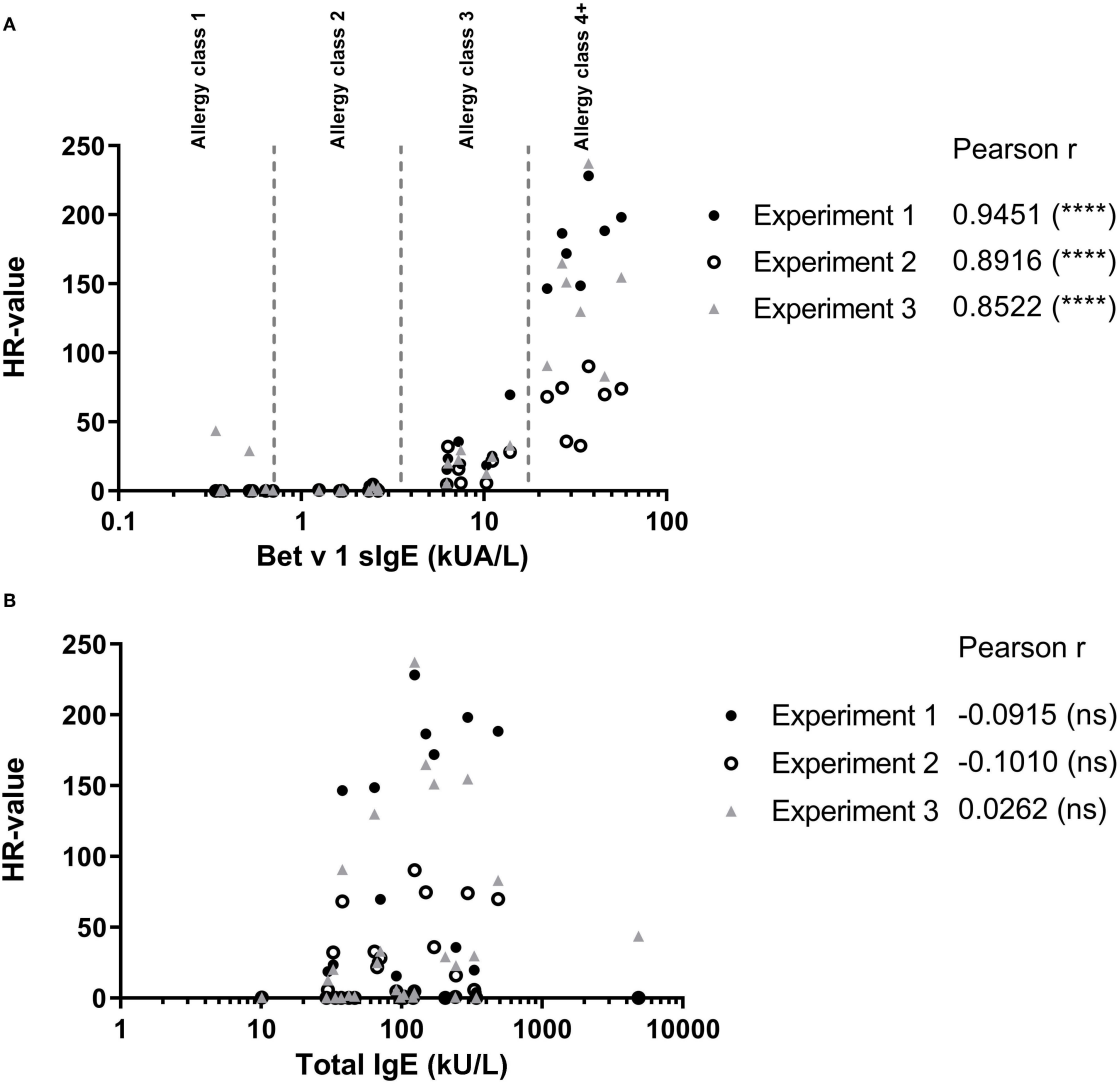
**(A)** Results from the passive HR (expressed as HR-value) and the Bet v 1 sIgE concentration in serum used for passive sensitization. **(B)** HR-value plotted against the total IgE concentration in serum. • Experiment 1; ◦ Experiment 2; and ▴ Experiment 3. Allergy classes are indicated in **(A)**. Pearson correlation.

**Figure 3:**
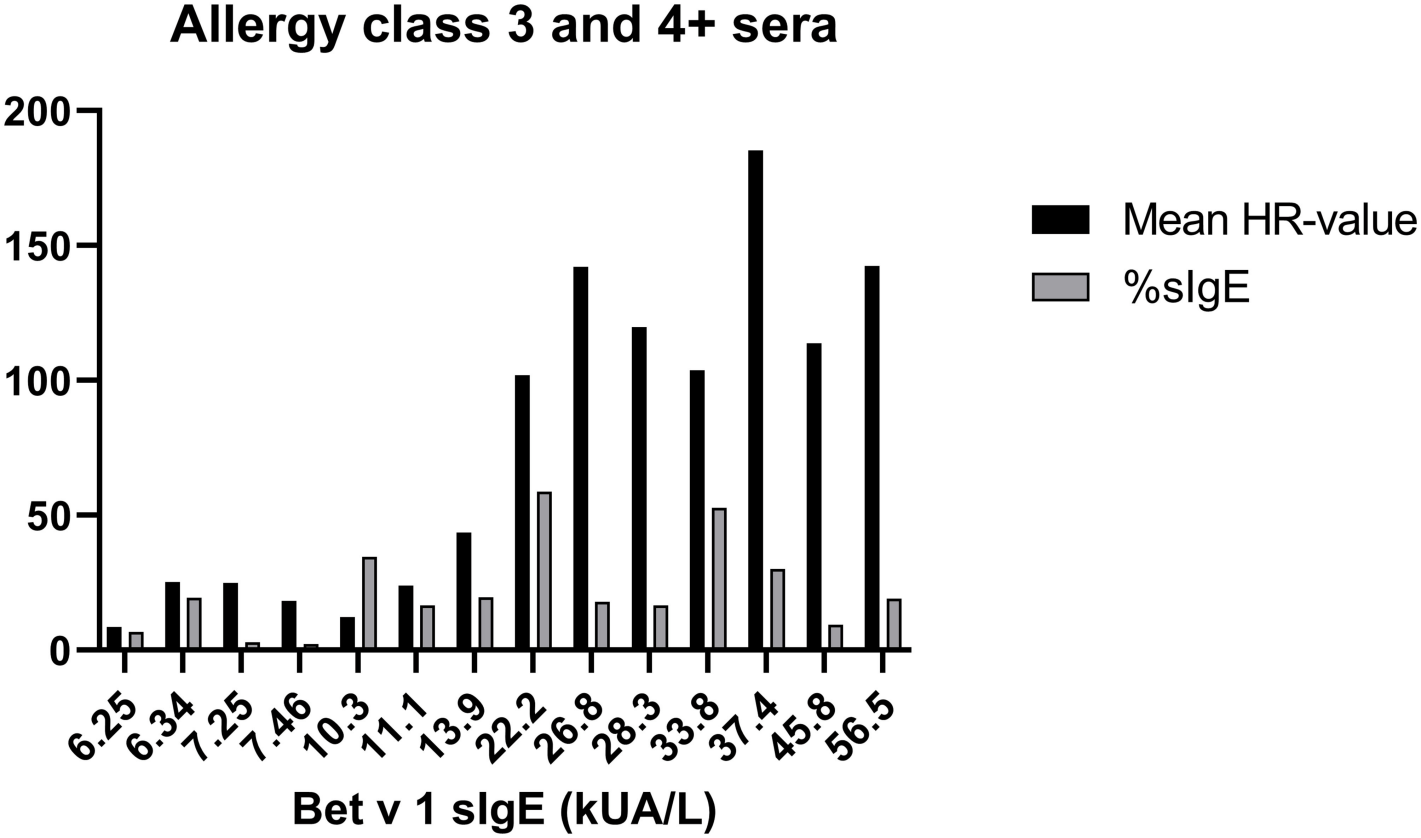
Data from sera in allergy classes 3 and 4+ are shown; Bet v 1 concentration (kU/l) at the x-axes, mean of the HR-value from the 3 experiments in black column, % of Bet v 1 sIgE (%sIgE) in gray column.

To evaluate how the level of sIgE affects basophil HR using PS, the HR values were plotted against the level of sIgE and as shown in Figure 2A, the correlation is good. No correlation was found using total IgE (Figure 2B). Thus, a stronger HR is observed from basophils when using sera with higher concentrations of sIgE. Nevertheless, zooming in on the individual classes the correlation is less clear. Using results from the allergy classes 3 and 4+ sera, it is notable that the HR value does not follow the level of sIgE (Figure 3). This dissociation between sIgE and the HR value cannot be explained by the % of Bet v 1 sIgE out of total IgE (Figure 3; Table 1).

### Histamine Release Induced by Matrix-Fixed Allergen

**Figure 4:**
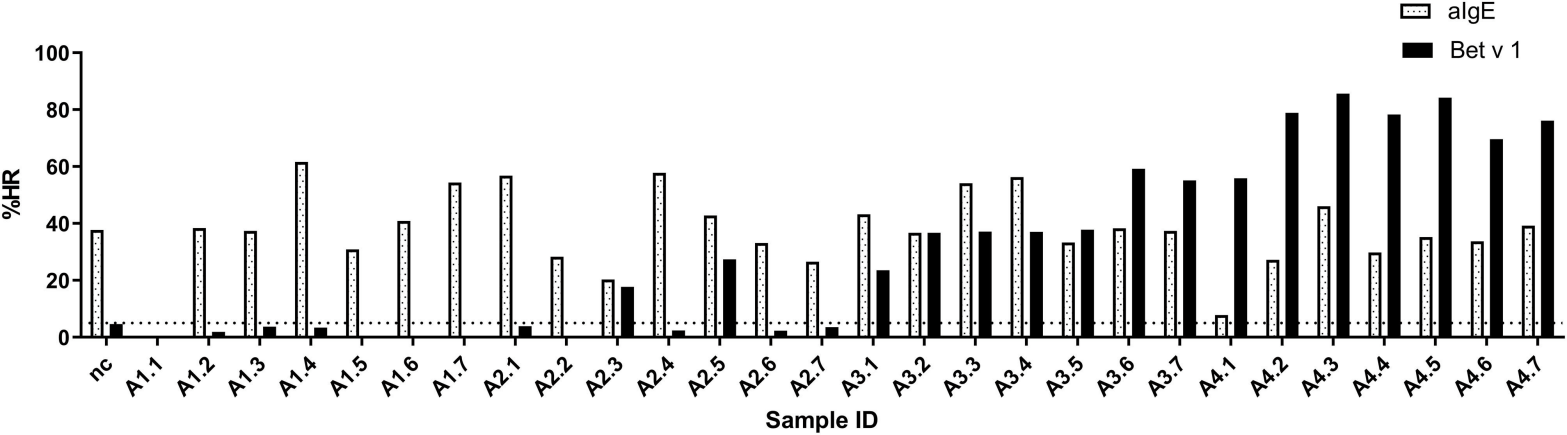
PS basophils activated by matrix fixed allergen using Bet v 1 or anti-IgE (assay control) ImmunoCAP™. Released histamine was extracted and quantified. Dotted horizontal line at 5% indicates background; nc, negative control.

**Figure 5:**
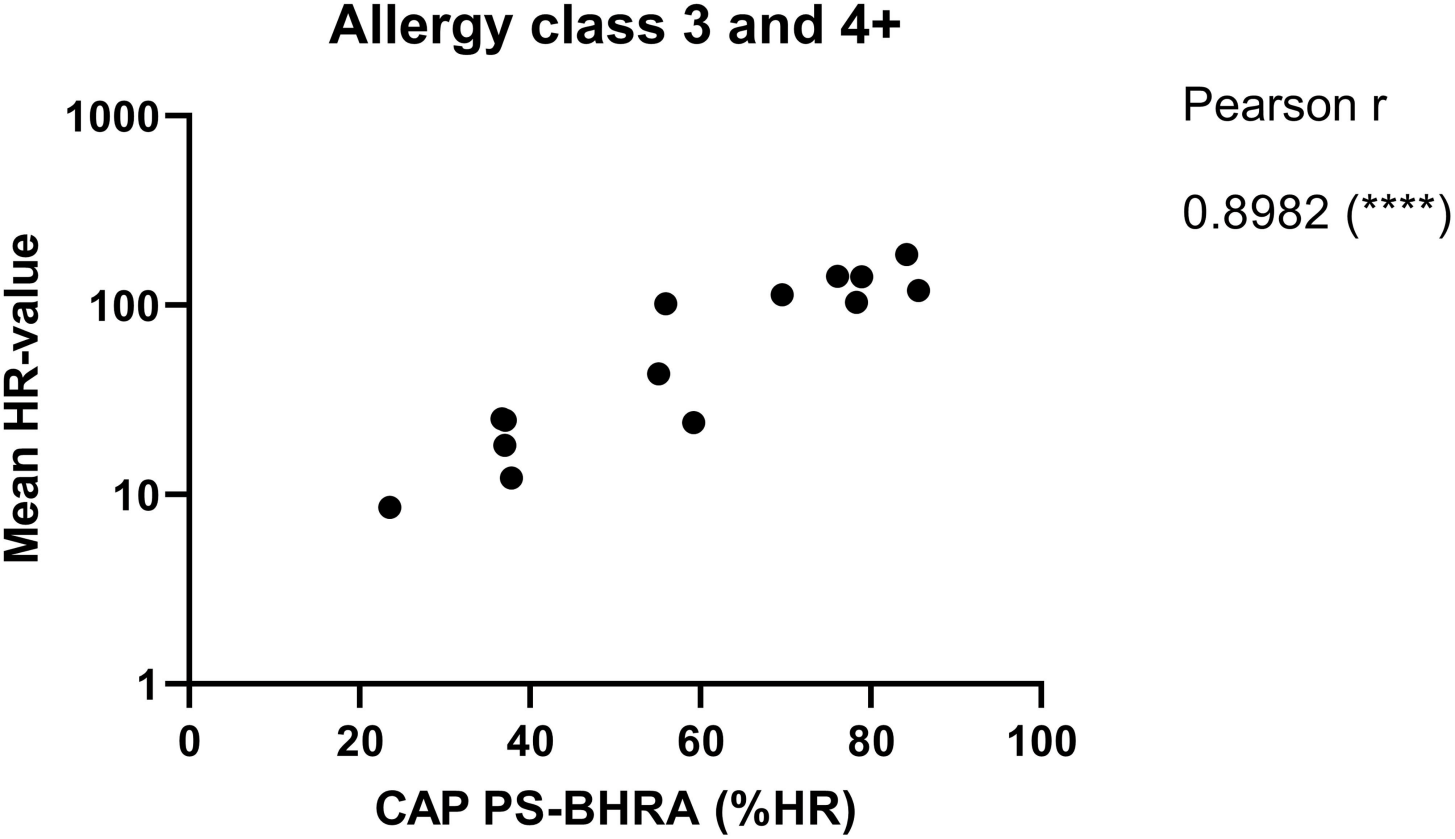
Mean of the HR-value from the 3 experiments correlated with PS basophils activated by matrix fixed allergen (CAP PS-BHRA) for the allergy classes 3 and 4+ sera. Pearson correlation.

The ImmunoCAP™ system holds more than 500 allergen tests and it would, therefore, be a huge advantage if these matrix-fixed allergens could be used as an allergen source if they produce an HR similar to a soluble allergen. We used the 28 sera for PS of basophils which were then loaded in either Bet v 1 or anti-IgE containing ImmunoCAP™s and released histamine was extracted and quantified. The anti-IgE ImmunoCAP™s were used as an assay control to prove the reactivity of the basophils if the Bet v 1 response was absent. As seen from Figure 4 and Supplementary Figure S1, a significant correlation was found (*p* = 0.9298) as Bet v 1 mediated HR is increasing with an increasing concentration of sIgE comparable with the results shown in Figures 1, 2. No HR was found with the allergy class 1 sera, 2/7 of allergy class 2 where the lowest sIgE concentration mediating an HR was 1.68 kUA/L, and 7/7 for both allergy classes 3 and 4+. We, therefore, did not gain more sensitivity using the same allergen system by which the IgE was quantified. However, using sera in allergy classes 3 and 4+ it seems likely that a single concentration of matrix-fixed allergen can be used equally to serial dilutions of soluble allergen as the results obtained with PS-BHRA (HR-value) correlate with the CAP PS-BHRA (Figure 5).

## Discussion

Passive sensitization of basophils may be used as a tool in allergy diagnosis when no sIgE test exists, fresh blood samples cannot be provided, or if the patient has non-releasing basophils (5). However, the real strength of PS lies within allergen characterization as PS takes into account both the IgE diversity using different sera, but keeps the cellular response uniform, which is useful, e.g., when screening for allergen candidates for immunotherapy (14). In 1977, MC Conroy et al. published that cell-bound IgE correlated with the IgE serum level; nevertheless, the releasability of the basophils was not entirely controlled by the IgE load as different donor basophils having approximately the same amount of IgE bound would respond differently to anti-IgE stimulation (27). Using passively sensitized basophils, this interdonor variation introduced by the cellular component is avoided as cells from one selected blood donor will provide basophils enough for multiple investigations. However, it is important to ensure optimal performance of the basophils which is why screening of blood donors is crucial for a successful PS. We used the criteria of more than 50 ng/ml histamine released (>30% release) after anti-IgE stimulation to ensure responding basophils (eliminating donors with non-releasing basophils) and a strong readout even if blood stored overnight would lose some activity. As the detection limit of the HR assay is 10 ng/ml, a 50% reduction of a 50 ng/ml HR response would still provide a feasible readout; however, the trend was more in the direction of an increase in the HR after storing the blood overnight, probably due to the addition of interleukin-3 (IL-3), which benefit basophil reactivity.

Another important aspect to consider before comparing HR data is in what way the HR data should be evaluated, as it often is given as a titration curve. We, therefore, introduced the HR-value to convert the full HR titration curve into a single number which considers both the location on the x-axis (sensitivity) and the y-axis (reactivity). Other parameters for sensitivity ($\frac{1}{2}$ max HR/ED50/CDsens) and reactivity (Ymax/CDmax) have previously been used (28–30). In addition, a traditional area under the curve (AUC) calculation has also been suggested as a readout to compare basophil activity or quantification of allergen in serum (17). However, these curve parameters are often challenged, e.g., when weak responses might have a $\frac{1}{2}$ max HR below the background (like allergy class 2 sera) or when the maximal response is not achieved (e.g., in allergy class 3). Furthermore, the sensitivity (the smallest allergen concentration needed to elicit an HR) is often omitted (AUC). Therefore, to evaluate the performance of the HR-value, we investigated how well AUC, CDsens, and Ymax correlated with sIgE and total IgE (Supplementary Table S2) and found that the Pearson correlation coefficient for sIgE was best with CDsens > AUC > Ymax, but that the HR-value was overall better than CDsens. None of the curve parameters correlated with total IgE. In addition, when looking at the number of tests that could be quantified, CDsens was poorer compared to AUC and Ymax, which performed equally well together with the HR-value (Supplementary Table S3). Therefore, taking the above data into account, we suggest using the HR-value when comparing dose-response curves as it embraces both the sensitivity and reactivity and has the best success in quantifying the response.

The challenge of PS is allocated to the selection of sera as not all the sera perform uniformly, i.e., result in lgE-mediated activation of basophils. In this study, we tested 28 different sera distributed among allergy classes 1 to 4+ and found that the lower limit of sIgE to mediate a positive response in the PS-BHRA was 1.25 kUA/L (allergy class 2) even though few sera with less sIgE were found to sensitize 1 or 2 of the cell donors. Among the allergy class 1 sera, 5 resulted in weak sensitization in experiment 3. The background was very low in this experiment and if we had used the mean background of all the 3 experiments, only serum A1.4 would appear positive which also is the serum giving a borderline response in experiment 1. On the other hand, experiment 2 had a high background causing a lower HR-value in this run. Despite this variation, which cannot be avoided due to donor-donor variance, the overall pattern was similar in the HR-values among the 3 experiments (Table 1).

Looking at different studies using PS (Supplementary Table S1), it is notable that to elicit an HR, the sIgE concentration needs to be in the area of 1.25–3.5 kUA/L if human basophils are used but if the assay builds on RBL cells, which often is used as a basophil surrogate, the sIgE concentration needs to be higher (3.91–219 kUA/L) (22). New technologies have been used to improve the RBL system, e.g., by combining it with a luciferase reporter system. Nevertheless, as the serum has to be diluted at 1:100 only sera within the allergy classes 3 and 4+ can be used (23). To avoid serum cytotoxicity a suggestion has been made to heat the serum sample for 5 min at 56°C, however, due to the unstable nature of IgE at this temperature, this might change the success for sensitization (31). Lately, human mast cells, either blood-derived or the cell line LAD2, have been added to the PS toolbox. However, even though mast cells might be a more potent effector cell compared to the basophil, the sIgE concentration needed to perform PS is at the same level as seen for PS of basophils (Supplementary Table S1).

Using a pure system, with recombinant sIgE against Der p2, Christensen, LH et al. also find that sensitization proven by a positive basophil activation test (BAT) only takes place when the amount of recombinant sIgE is high enough (8–10 ng/ml = allergy class 3) (32). Therefore, no matter the type of the cellular system or the purity and source of sIgE, it seems that to obtain a successful PS you need sIgE in the range of allergy classes 3 to 4+ (Supplementary Table S1).

The performance of the BHRA has been challenged by the BAT as the BHRA might be less sensitive due to the limit in the quantification of histamine. Nevertheless, when performing PS the sIgE level still needs to be in the area of allergy classes 2 to 4+ when PS basophils are used for BAT (26, 33). Furthermore, we have experienced that incubating basophils with serum can affect the receptor expression of CD203c (unpublished data).

We introduced a matrix-fixed allergen system (ImmunoCAP™) which could have a positive impact on the IgE-allergen binding and, thereby, the cross-linking of FcεRI and activation of the basophils. In contrast, replacing a dose-response curve with only one point might compromise the results as dose-response curves often are very broad and bell-shaped (28). Nevertheless, the reaction pattern and level of sIgE needed for activation were comparable between the two assays (1.25 vs. 1.65 kUA/L), again illustrating the level of sIgE necessary for a successful PS but also questing the need for an allergen titration curve. However, if one dilution of a soluble allergen would mediate the same result as one concentration of matrix-fixed allergen has to be further investigated.

We saw a strong correlation between the concentration of Bet v 1 sIgE and the HR-value but zooming on the individual allergy classes, this correlation was less clear. This is not explained by the concentration of total IgE as the percentage of Bet v 1 sIgE does also not follow the HR pattern. It has been described that sera containing > 10% sIgE perform well in PS but such sera are very likely to be allocated in allergy classes 3 and 4+ (Table 1) (21). An explanation for differences in response when using sera with approximately the same sIgE concentration could be the IgE clonality and affinity which seems to affect both the reactivity and sensitivity (32).

The type of allergen used when performing PS might also play a role. We had chosen birch pollen allergy as a “one-dimensional” model system since it has a single major allergen, Bet v 1, in contrast to, e.g., peanut, where Ara h 2/6 or Ara h3/h3.02 cross-reactivities may complicate interpretation of results. In addition, by using a small molecule as Bet v 1 (17 kDa), the findings might also be applicable to larger proteins, including more epitopes (34). Furthermore, according to the studies listed in Supplementary Table S1, sera from grass, house dust mite, or food allergic patients also perform well in PS when the level of sIgE is within allergy classes 3 and 4+, indicating that a successful PS is independent of the allergen system.

Overall PS is a useful technique embracing both diagnostic and allergen investigations. The limitation is within the concentration of sIgE needed to perform optimal PS. This is important to address especially if PS is used in diagnosis as false negative results can appear when the sIgE level is too low. Therefore, the concentration of sIgE has to be evaluated before using PS.

## Conclusion

By using birch allergy and rBet v 1 as an allergen model system, we have demonstrated that the IgE titer is strikingly robust in predicting the ability to sensitize basophils and produce a measurable HR no matter if the allergen is in suspension or fixed to a matrix. We believe our results strengthen the selection of sera for future studies discriminating the ones with low potential for success in passive sensitization.

## Data Availability Statement

The original contributions presented in the study are included in the article/Supplementary Material, further inquiries can be directed to the corresponding author/s.

## Ethics Statement

The studies involving human participants were reviewed and approved by De Videnskabsetiske Komiteer for Region Hovedstaden, protocol H-3-2010-090. Written informed consent for participation was not required for this study in accordance with the national legislation and the institutional requirements.

## Author Contributions

BJ, PSS, and LP contributed to conceptualization and supervision. PS, BJ, and LP contributed to formal analysis. PS and BJ investigated the study, wrote the original draft, and visualized the study. LP contributed to resources. BJ contributed to project administration. All the authors were involved in validation, methodology, writing-review and editing, and have read and agreed to the published version of the manuscript.

## Funding

This study was supported by the Novo Scholarship Program.

## Conflict of Interest

PSS is head of research at RefLab. The remaining authors declare that the research was conducted in the absence of any commercial or financial relationships that could be construed as a potential conflict of interest.

## Publisher's Note

All claims expressed in this article are solely those of the authors and do not necessarily represent those of their affiliated organizations, or those of the publisher, the editors and the reviewers. Any product that may be evaluated in this article, or claim that may be made by its manufacturer, is not guaranteed or endorsed by the publisher.

## Supplementary Material

Supplementary Figure S1 Results from PS basophils activated by matrix fixed allergen using ImmunoCAP™ (CAP PS-BHRA) and the log concentration of Bet v 1 specific IgE in serum used for passive sensitization. • Experiment 1; ◦ Experiment 2; and ▴ Experiment 3. Allergy classes are indicated. Pearson correlation.

Supplementary Table S1 Overview of passive sensitization models and the sIgE concentration in the serum sample eliciting a response.

Supplementary Table S2 Pearson correlation coefficients between serological parameters and various titration curve algorithms.

Supplementary Table S3 Number of quantitative tests within each allergy class when using different curve titration curve algorithms.
