# Supplementary figures and images for: The Allergen-Specific IgE Concentration Is Important for Optimal Histamine Release From Passively Sensitized Basophils
Source: Front Allergy. 2022 Apr 7;3:875119. doi: 10.3389/falgy.2022.875119 (PMC9234936; doi:10.3389/falgy.2022.875119)

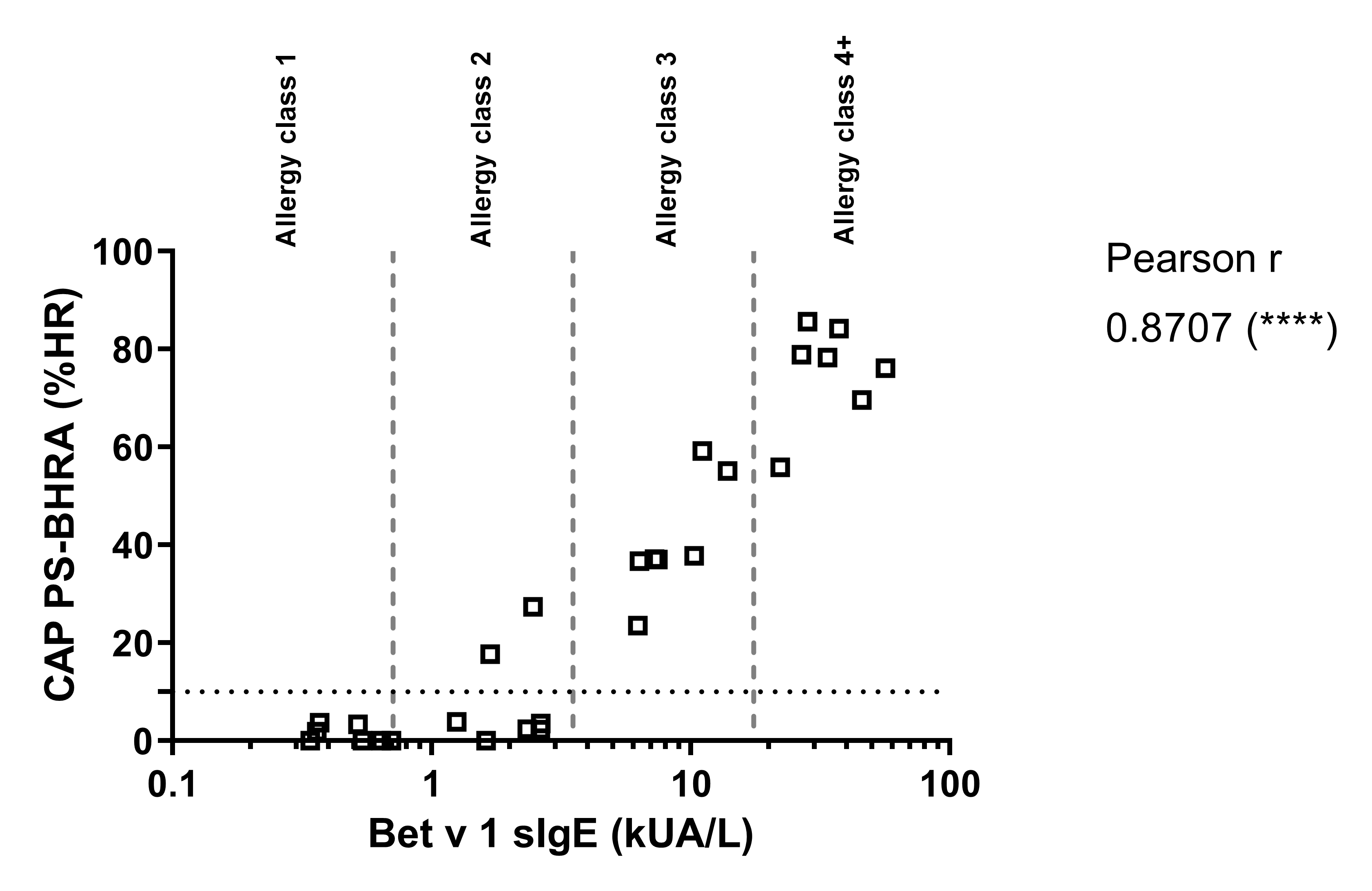

Supplement: Supplementary Figure S1 — Results from PS basophils activated by matrix fixed allergen using ImmunoCAP™ (CAP PS-BHRA) and the log concentration of Bet v 1 specific IgE in serum used for passive sensitization. • Experiment 1; ◦ Experiment 2; and ▴ Experiment 3. Allergy classes are indicated. Pearson correlation. [file Image_1.TIF]
