# Supplementary material for: The Allergen-Specific IgE Concentration Is Important for Optimal Histamine Release From Passively Sensitized Basophils
Source: Front Allergy. 2022 Apr 7;3:875119. doi: 10.3389/falgy.2022.875119 (PMC9234936; doi:10.3389/falgy.2022.875119)
Supplement: Supplementary Table S1 — Overview of passive sensitization models and the sIgE concentration in the serum sample eliciting a response. [file Table_1.DOCX]

**Table S1**: Overview of passive sensitization models and the sIgE concentration in the serum sample eliciting a response.

| Cell model | Allergen | Read out | Serum conc. | sIgE | Reference |
| --- | --- | --- | --- | --- | --- |
| Human basophils | Bet v1 | HR | 11% | 1.25 kU/L  (> 6.25 kU/L  = 100% response) | Jensen,BM et al. |
| Human basophils | Peanut | BAT | ? | ≥2.26 KU_A_/L * | (33) |
| Human basophils | rDer p2 | BAT | srIgE | 8-10 ng/ml ** | (32) |
| Human basophils | HDM | HR | 15% | >2.7 IU/ml | (35) |
| Human basophils | Food | BAT | ? | >3.5 kU/L | (26) |
| RBL-FcεRI transfected | Egg white protein | Luciferase reporter gene | 1% | >3.91 U_A_/L ¤ | (23) |
| RBL-FcεRI transfected | Peanut extract | β-hexosaminidase | 4-10% | >36 kU/L § | (19) |
| RBL-FcεRI transfected | Birch pollen  Timothy | β-hexosaminidase | 2.5-5% | >41 kU/L  >9 kU/L | (20) |
| RBL-FcεRI transfected | Peanut extract | Serotonin | 11% | >10 IU/L | (21) |
| RBL-FcεRI transfected | Dactylis glomerata,  Der p  extracts | β-hexosaminidase | 2% | >60 kU/L  >219 kU/L $ | (22) |
| Mouse MC FεcRI-transfected | Peanut, Cat, Wasp, Honey bee, Dust mite, Birch pollen,  Timothy | Hoxb8 | ? | < 6.34 kU/L | (25) |
| Human MC  (PB derived) | Peanut | CD63 | 10% | 4.3 kU/L | (24) |
| Human MC (LAD2) | Peanut | CD63 | ? | ≥2.26 KU_A_/L * | (33) |

PB: Peripheral Blood

*) No real cut-off concentration is reported, only that sera from peanut allergics (IQR: 2.26-98.70KU_A_/L) were responsive in PS whereas sera from peanut sensitized but tolerant subjects (IQR: 0.48-5.20KU_A_/L) were negative in PS.

**) Correspond to 3.3-4.4 kU/L.

¤) One serum sample with sIgE at 4.46 U_A_/L was found unresponsive.

§) One serum sample with sIgE for peanut > 100kU/L was found only to give a borderline response.

$) One serum sample with a sIgE for Der p on 74 kU/L gave a borderline response whereas two sera at 83 and 84kU/L did not elicitate a response.
