# Supplementary material for: The Allergen-Specific IgE Concentration Is Important for Optimal Histamine Release From Passively Sensitized Basophils
Source: Front Allergy. 2022 Apr 7;3:875119. doi: 10.3389/falgy.2022.875119 (PMC9234936; doi:10.3389/falgy.2022.875119)
Supplement: Supplementary Table S2 — Pearson correlation coefficients between serological parameters and various titration curve algorithms. [file Table_2.DOCX]

**Table S2:** Pearson correlation coefficients between serological parameters and various titration curve algorithms.

| **Serology** | **Experiment** | **HR-value** | **AUC** | **CDsens** | **Ymax** |
| --- | --- | --- | --- | --- | --- |
| sIgE (kU/L) | 1 | 0.9451 | 0.8161 | 0.9385 | 0.7982 |
|  | 2 | 0.8916 | 0.8119 | 0.8896 | 0.7938 |
|  | 3 | 0.8522 | 0.8496 | 0.8954 | 0.7988 |
| Total IgE (kU/L) | 1 | -0.0915 | -0.1560 | -0.1002 | -0.1784 |
|  | 2 | -0.1010 | -0.1512 | -0.1207 | -0.1744 |
|  | 3 | 0.0262 | -0.1572 | -0.1096 | -0.1587 |
