# Supplementary material for: The Allergen-Specific IgE Concentration Is Important for Optimal Histamine Release From Passively Sensitized Basophils
Source: Front Allergy. 2022 Apr 7;3:875119. doi: 10.3389/falgy.2022.875119 (PMC9234936; doi:10.3389/falgy.2022.875119)
Supplement: Supplementary Table S3 — Number of quantitative tests within each allergy class when using different curve titration curve algorithms. [file Table_3.DOCX]

**Table S3:** Number of quantitative tests within each allergy class when using different curve titration curve algorithms.

| **Curve Parameter** | **Allergy class 1** | **Allergy class 2** | **Allergy class 3** | **Allergy class 4** |
| --- | --- | --- | --- | --- |
| **HR-value** | 5 / 21 | 15 / 21 | 21 / 21 | 21 / 21 |
| **AUC** | 5 / 21 | 15 / 21 | 21 / 21 | 21 / 21 |
| **CDsens** | 0 / 21 | 6 / 21 | 21 / 21 | 21 / 21 |
| **Ymax** | 5 / 21 | 15 / 21 | 21 / 21 | 21 / 21 |
